# Mediating role of growth mindset between college life stress and adjustment to college life among nursing students: A-cross sectional study

**DOI:** 10.1371/journal.pone.0325774

**Published:** 2025-06-25

**Authors:** Sujin Kang, Hye-Ah Yeom

**Affiliations:** 1 College of Nursing, The Catholic University of Korea, Seoul, Korea; Aalborg University, DENMARK

## Abstract

Adjusting to college life is important for nursing students to achieve academic and work performance successfully. Growth mindset has been proposed as a mediating concept that can induce positive psychological outcomes in stressful life contexts, and may be a potential facilitating factor for adjustment to college life among nursing students experiencing stressful college life. This study aimed to examine the mediating effect of growth mindset in the relationship between college life stress and adjustment to college life in nursing students. This was a cross-sectional descriptive study. The study participants were 250 undergraduate nursing students recruited from three nursing colleges in Korea. Self-reported data on college life stress, growth mindset and adjustment to college life were collected using the online survey questionnaire. The data were analyzed using an independent t-test, one-way ANOVA, Kruskal-Wallis H test, Pearson’s correlation, and multiple regression analysis. The mediating effects were verified through the bootstrapping method using the PROCESS macro for SPSS. Higher levels of adjustment to college life were reported in nursing students who were in good health status and were satisfied with the nursing major. Nursing students’ adjustment to college life was negatively affected by college life stress and positively affected by growth mindset. Growth mindset mediated the link between college life stress and adjustment to college life of nursing students. Educators may be aware that growth mindset can transform stress into challenging opportunities for nursing students. As both types of growth mindset—intelligence and personality—mediated the relationship between college life stress and adjustment to college life, pedagogical strategies cultivating growth mindset in both domains should be developed and implemented to support nursing students’ adjustment.

## Introduction

College students are in the stage of transitioning from adolescence to adulthood, and may experience significant stress while adjusting to the rapidly changing social environment in college life [1]. They face stress from multiple sources, including academic pressure, financial burdens, concerns about meeting parental expectations, building interpersonal relationships, and adjusting to new environments [2,3]. College students experiencing higher levels of stress are known to exhibit poorer adjustment outcomes, such as a higher risk of mental health problems and decreased academic engagement [4,5]. In the case of nursing students, they experience a high level of psychological stress that is induced from learning tasks related to their major, including taking theory courses, clinical practice, and preparation for the national licensing examination for registered nurses [6]. Particularly, academic achievement is a main source of stress for nursing students [7]; the degree of stress related to academic activities is known to be higher in nursing students [8,9]. Thus, it is important for nursing students to appropriately manage their psychological stress to successfully adjust to college life.

Generally, adjustment to college life affects academic adaptation, satisfaction with the major, learning activities, and employment for college students [10]. During their college years, nursing students acquire basic knowledge on human health and learn professional nursing roles to prepare for complex clinical contexts as future nurses [11]. Inadequate adjustment to college life can result in the student’s withdrawal from a nursing major, or it may have negative long-term impacts on their adjustment to work life as a newly graduated nurse [12]. For nursing students, adjustment to college life may impact on their work life even after graduation [13] and clinical competence in the future [14]. Therefore, adjusting to college life is an important matter for nursing students and its influencing factors should be identified to develop interventions for facilitating their adjustment to college life.

Implicit theories refer to individuals’ beliefs about the extent to which traits such as ability, personality, or intelligence can change. These beliefs can influence a person’s motivation, behavior, and learning outcomes. The theory includes two main perspectives: the growth mindset and the fixed mindset [15]. Growth mindset refers to the belief that an individual’s abilities and intelligence can be improved through personal experiences and efforts [16]; this is the opposite notion of a fixed mindset, which refers to the view that a person’s competence is fixed and unchangeable regardless of the degree to which the person strives to change. The growth mindset can be divided into two subdomains: ‘intelligence’ and ‘personality’. It reflects the belief that intelligence and personality are not fixed traits but can be developed through effort and learning [16]. A growth mindset of intelligence has been shown to have a positive impact on academic achievement [17–19], and has been found to influence personal well-being, such as stress regulation and mental health [19,20]. A growth mindset of personality is known to enhance self-efficacy in interpersonal relationships, help individuals cope effectively with social stress, and protect them from maladaptive coping and psychological distress [21]. Growth mindset acts as a certain tendency that a person exhibits in thinking and interpreting a situation [22], and it is linked to the direction of a goal [15]. Individuals with a growth mindset tend to perceive stressful situations as opportunities for personal growth and believe that they can overcome life challenges through their own efforts [22]. Growth mindset can convert stress into positive motivational stimuli, leading the individual to make more effective adjustments to their current contexts, and has been proposed as a mediating concept that can induce positive psychological outcomes, happiness, or life adjustment in stressful life contexts [23–25].

Previous research revealed that increased levels of growth mindset are associated with decreased psychological distress [26,27] and increased coping strategies [27]. According to implicit theories, growth mindset improves mental health by promoting adaptive coping strategies (e.g., persistence in challenges) and reducing helplessness in stress-inducing situations [15,16]. For example, single-session interventions (SSIs) targeting growth mindset of intelligence have been reported to alleviate anxiety and promote psychological well-being, positive self-evaluation [19], and academic achievement [17,19]. SSIs targeting growth mindset of personality have also been reported to reduce anxiety, depression [28] and hopelessness, self-hate, and perception of control [29]. The distinctive roles of growth mindset of intelligence and growth mindset of personality should be recognized; the role of growth mindset of intelligence includes enhancing academic resilience and mitigating the negative effects of academic stress on performance [15,18], whereas the role of growth mindset of personality includes improving emotional regulation and buffering psychosocial stressors (e.g., social rejection, self-criticism) [18,26,27].

The present study was based on the notion that growth mindset is a potential facilitating factor for adjustment to college life among nursing students experiencing stressful college life. Therefore, this study aimed to investigate how growth mindset can influence the relationship between a nursing student’s college life stress and their adjustment to college life. The objectives of this study are as follows: (1) To examine the characteristics including demographics (gender, age, grade, religion, type of residence, perceived health status, reason for majoring in nursing, satisfaction with the nursing major, focus of high school curriculum, and participation in club activities) of nursing students. (2) To measure the levels of college life stress, growth mindset, and adjustment to college life among nursing students and explore their respective subdomains. (3) To investigate the differences in college life stress, growth mindset, and adjustment to college life according to the participants’ characteristics including demographics. (4) To analyze the correlations among college life stress, growth mindset, and adjustment to college life. (5) To examine the mediating effect of growth mindset on the relationship between college life stress and adjustment to college life among nursing students. The specific hypotheses based on the objectives of the study are as follows:

1. College life stress will have a negative correlation with growth mindset (intelligence and personality).
2. Growth mindset (intelligence and personality) will have a positive correlation with adjustment to college life.
3. Growth mindset (intelligence and personality) will mediate the relationship between college life stress and adjustment to college life by mitigating the negative impact of stress.

## Methods

### Participants

The study participants were 250 nursing students who signed a consent form that explains the objectives of this study clearly. Only students who had been in college for more than one year were included as the study participants. The sample size was calculated using the G-Power 3.1 program based on a significance level of.05, a power of.95, an effect size of.15, and 13 predictive factors for multiple regression analysis. This resulted in 189 participants as a minimum number of subjects. The final number of subjects included 250 nursing students after the consideration of a 30% of potential dropout rate.

### Design and setting

This study used a cross-sectional descriptive study design. The study participants were recruited from three nursing colleges in South Korea using a convenience sampling method.

### Data collection procedure

The data were collected from September to November, 2023. The participants were recruited through online communities of three nursing colleges. The researchers posted online recruitment flyers that described the research purpose, participant inclusion criteria, and benefits and risks related to study participation. Participants were recruited on a voluntary basis, and those who were interested in study participation completed the online survey questionnaire. Approximately 10–15 minutes were spent on the completion of the survey. Participants were compensated with a gift voucher for their participation.

### Ethical considerations

This study was approved by the Institutional Review Board of the C University (approval number MC23QASI0073). All participants signed a written informed consent after being explained of the objectives of the study. Participants were assured that the contents of the survey would be used solely for research purposes and all questionnaires would be processed anonymously.

### Measurements

#### Characteristics including demographics.

The participants’ characteristics, including demographics, were assessed using ten questions: gender, age, grade, religion, type of residence, perceived health status, reason for majoring in nursing, satisfaction with the nursing major, focus of high school curriculum (natural sciences, humanities or others), and participation in club activities. Each item consisted of a single question. Age was recorded as an open-ended response in full years, while the remaining items were multiple-choice. Religion was measured in a dichotomous way in which whether an individual has religion or not, and there was no assessment of the type of religion for those who reported they had a religious affiliation. Participants were asked to indicate the focus of their high school curriculum (humanities, natural sciences, or other) to examine whether their academic interest and background during high school years were related to their choice of nursing as a college major. These variables and their corresponding response categories were determined based on previous studies [9,11,13,30–32] that identified them as relevant factors for college life adjustment.

#### College life stress.

College life stress was measured using the Revised Life Stress Scale for College Students (RLSS-CS) developed by Chon et al. [33] and revised by Lee [34]. The 50-item scale measures the frequency of stress experiences over the past year and is composed of two subdomains: interpersonal relationships and task-related stress. The domain of interpersonal relationships consists of items related to relationships with friend, relationships with lover, relationships with family, and relationships with faculty. The domain of task-related stress consists of items related to academic problems, economic problems, future problems, and value problems. Each item is measured on a 5-point Likert scale ranging from 1 (*not stressed at all*) to 5 (*stressed very often*). A higher score indicated a higher degree of stress in college life. The Cronbach’s alpha reliability of the scale was.94 in this study.

#### Growth mindset.

Growth mindset was measured using a scale developed by Dweck [16] and translated into Korean by Lee et al. [35]. The growth mindset scale is composed of eight items with two subdomains: intelligence (beliefs about changes in intelligence) and personality (beliefs about changes in personality). Each question is measured on a 5-point Likert scale ranging from 1 (*strongly disagree*) to 5 *(strongly agree*), with higher scores indicating higher levels of growth mindset. The scale uses a sum score, with higher total scores indicating higher general levels of growth mindset. The sum scores of subdomains, including growth mindset of intelligence and growth mindset of personality, were also calculated to assess the levels of each growth mindset type. Reverse coding was conducted for 4 items. In this study, the Cronbach’s alpha reliability of the scale was.82.

#### Adjustment to college life.

Adjustment to college life was measured using the College Adjustment Scale (CAS) developed by Jeong and Park [36]. The CAS consists of 19 questions with five subdomains: interpersonal relationships, academic activities, career preparation, personal psychology, and social experiences. Each item is measured on a 5-point Likert scale, ranging from 1 *(strongly disagree*) to 5 (*strongly agree*). Higher scores indicated higher levels of adjustment to college life. The Cronbach’s alpha reliability of the scale was.88 in this study.

#### Data analysis.

The data were analyzed using IBM SPSS Statistics version 26.0 and SPSS PROCESS macro version 4.2 [37]. The significance level (alpha) was set at 0.05 for all statistical tests. Descriptive statistics were estimated for all study variables. The differences in college life stress, growth mindset, and adjustment to college life according to characteristics including demographics were analyzed using an independent t-test, one-way analysis of variance (ANOVA), and Kruskal-Wallis H test. The Kruskal-Wallis H test, a non-parametric alternative to one-way ANOVA, was employed because the assumption of normality was violated in some groups. The correlations among college life stress, growth mindset, and adjustment to college life were examined using Pearson’s correlation coefficients. For regression analysis, multi-collinearity between independent variables was checked using tolerance and the variance inflation factor (VIF). Autocorrelation of the dependent variable was tested using Durbin-Watson values. The mediating effect was examined using multiple regression analysis. Bootstrapping was used to test the statistical significance of the indirect effect of the mediator. The number of re-sampling times for bootstrapping was set to 5,000, and a 95% confidence interval was estimated.

## Results

### Participants’ characteristics including demographics

Most participants were female (83.6%) and the mean age of the participants was 22 years (*SD* = 2.15). Approximately half of the participants were sophomores (43.6%) and living alone (46.8%). One third of the participants (36.4%) had religion. The majority of the participants perceived their health status as moderate (n=96, 38.4%) or good (n=96, 38.4%). The most common reason for majoring in nursing was interest in a nursing major (n=102, 40.8%) and job security (n=84, 33.6%). In terms of satisfaction with the nursing major, the majority of the nursing students reported that they were satisfied (n=129, 51.6%) or very satisfied with their major (n=24, 9.6%) (Table 1).

**Table 1:** College life stress, growth mindset, adjustment to college life in nursing students by characteristics including demographics (*N* = 250).

Variables	N (%)	College life stress	Growth mindset	Growth mindset of intelligence	Growth mindset of personality	Adjustment to college life
		M ± SD	M ± SD	M ± SD	M ± SD	M ± SD
		t or F (*p*)	t or F (*p*)	t or F (*p*)	t or F (*p*)	t or F (*p*)
Gender						
Female	209 (83.6%)	2.35 ± 0.56	3.07 ± 0.71	3.16 ± 0.85	2.98 ± 0.84	3.41 ± 0.57
Male	41 (16.4%)	2.36 ± 0.59	3.27 ± 0.72	3.40 ± 0.83	3.13 ± 0.89	3.38 ± 0.59
		−0.09(.931)	−1.63(.105)	−1.64(.102)	−1.10(.274)	0.26(.798)
Grade						
2nd ^a^	109 (43.6%)	2.43 ± 0.54	3.07 ± 0.76	3.22 ± 0.89	2.91 ± 0.84	3.28 ± 0.52
3rd ^b^	82 (32.8%)	2.36 ± 0.58	3.15 ± 0.73	3.17 ± 0.88	3.13 ± 0.88	3.39 ± 0.60
4th ^c^	59 (23.6%)	2.21 ± 0.59	3.08 ± 0.62	3.19 ± 0.74	2.98 ± 0.82	3.64 ± 0.56
		2.62(.075)	0.34(.709)	0.10(.904)	1.64(.195)	7.98(<.001) a,b < c^†^
Religion						
No	159 (63.6%)	2.39 ± 0.57	3.06 ± 0.72	3.13 ± 0.84	2.98 ± 0.86	3.34 ± 0.55
Yes	91 (36.4%)	2.29 ± 0.57	3.18 ± 0.71	3.31 ± 0.86	3.04 ± 0.83	3.51 ± 0.60
		−1.32(.190)	1.28(.202)	1.63(.104)	0.53(.599)	2.24(.026)
Type of residence						
Dormitory	58 (23.2%)	2.41 ± 0.48	3.00 ± 0.75	3.02 ± 0.88	2.97 ± 0.84	3.38 ± 0.55
Living alone	117 (46.8%)	2.37 ± 0.61	3.13 ± 0.69	3.28 ± 0.83	2.97 ± 0.86	3.37 ± 0.55
Living with parents	63 (25.2%)	2.28 ± 0.56	3.15 ± 0.72	3.18 ± 0.83	3.11 ± 0.85	3.48 ± 0.64
Others	12 (4.8%)	2.36 ± 0.63	3.08 ± 0.77	3.33 ± 0.94	2.81 ± 0.82	3.38 ± 0.56
		1.73^*^(.629)	1.94^*^(.585)	3.77^*^(287)	1.91^*^(.592)	0.57(.635)
Perceived health status						
Very poor ^a^	3 (1.2%)	3.17 ± 0.13	2.92 ± 0.40	3.25 ± 0.25	2.58 ± 0.80	3.18 ± 0.74
Poor ^b^	33 (13.2%)	2.62 ± 0.35	2.90 ± 0.73	2.93 ± 0.91	2.86 ± 0.82	3.06 ± 0.65
Moderate ^c^	96 (38.4%)	2.33 ± 0.60	3.11 ± 0.73	3.16 ± 0.88	3.05 ± 0.88	3.36 ± 0.57
Good ^d^	96 (38.4%)	2.34 ± 0.54	3.11 ± 0.69	3.27 ± 0.78	2.94 ± 0.84	3.48 ± 0.50
Very good ^e^	22 (8.8%)	2.06 ± 0.63	3.36 ± 0.72	3.43 ± 0.88	3.30 ± 0.80	3.80 ± 0.44
		24.75^*^(<.001)	6.82^*^(.146)	6.46^*^(.167)	5.66^*^(.226)	7.02(<.001)
		a, b > e^‡^ b > c, d^‡^				b < d, e^†^ c < e^†^
Reason for majoring in nursing						
Voluntary motivation	102 (40.8%)	2.30 ± 0.58	3.17 ± 0.68	3.28 ± 0.84	3.07 ± 0.84	3.50 ± 0.54
Recommendations from parents, teachers, etc	39 (15.6%)	2.40 ± 0.60	2.95 ± 0.70	3.06 ± 0.83	2.83 ± 0.76	3.37 ± 0.61
Job security	84 (33.6%)	2.40 ± 0.53	3.12 ± 0.72	3.23 ± 0.84	3.00 ± 0.89	3.32 ± 0.60
High school grades	12 (4.8%)	2.42 ± 0.74	3.04 ± 0.74	2.96 ± 0.95	3.13 ± 0.81	3.32 ± 0.53
Good image of a nurse	9 (3.6%)	2.45 ± 0.52	2.90 ± 0.98	2.81 ± 0.97	3.00 ± 1.05	3.42 ± 0.64
Other reasons	4 (1.6%)	1.99 ± 0.10	3.03 ± 0.93	3.44 ± 0.97	2.63 ± 0.97	3.14 ± 0.31
		5.09^*^(.406)	0.73(.599)	5.58^*^(.349)	3.43^*^(.634)	1.15(.334)
Satisfaction with the nursing major						
Not satisfied at all ^a^	7 (2.8%)	2.81 ± 0.27	2.95 ± 0.52	3.21 ± 0.67	2.68 ± 0.43	2.44 ± 0.52
Unsatisfied ^b^	30 (12.0%)	2.79 ± 0.51	2.98 ± 0.60	2.98 ± 0.82	2.99 ± 0.71	3.20 ± 0.58
Moderate ^c^	60 (24.0%)	2.55 ± 0.53	3.07 ± 0.67	3.10 ± 0.81	3.04 ± 0.84	3.19 ± 0.52
Satisfied ^d^	129 (51.6%)	2.19 ± 0.50	3.11 ± 0.78	3.25 ± 0.89	2.97 ± 0.89	3.52 ± 0.48
Very Satisfied ^e^	24 (9.6%)	2.11 ± 0.66	3.29 ± 0.64	3.41 ± 0.73	3.18 ± 0.91	3.87 ± 0. 57
		13.32(<.001)	0.74(.563)	4.47^*^(.346)	2.23^*^(.693)	16.48(<.001)
		a b,c > e^†^ b,c > d^†^				a < b,c,d,e^†^ b < e^†^, c < d,e^†^
Focus of high school curriculum						
Natural sciences	149 (59.6%)	2.39 ± 0.54	3.04 ± 0.72	3.16 ± 0.85	2.92 ± 0.88	3.36 ± 0.54
Humanities	80 (32.0%)	2.33 ± 0.59	3.18 ± 0.67	3.23 ± 0.80	3.13 ± 0.79	3.44 ± 0.59
Others	21 (8.4%)	2.40 ± 0.51	3.21 ± 0.82	3.33 ± 1.02	3.10 ± 0.79	3.32 ± 0.58
		1.77^*^(.413)	1.25(.287)	0.55^*^(.759)	3.40^*^(.183)	0.71(.494)
Club activities						
No	146 (58.4%)	2.35 ± 0.63	3.07 ± 0.71	3.22 ± 0.84	2.92 ± 0.82	3.28 ± 0.51
Yes	104 (41.6%)	2.36 ± 0.52	3.14 ± 0.73	3.16 ± 0.86	3.11 ± 0.88	3.57 ± 0.62
		−0.18(.856)	0.68(.499)	−0.58(.562)	1.73(.085)	3.96(<.001)

M=mean; SD=standard deviation.

* Kruskal-Wallis H test.

† The results of Scheffé test.

‡ The results of Bonferroni correction method.

### Levels of college life stress, growth mindset, and adjustment to college life

The mean score for college life stress was 2.36 out of 5 points. The mean score for the domain of task-related stress (2.81) was higher than that for interpersonal relationships (1.79). In terms of task-related stress, the area with the highest mean stress score was academic problems (3.55). In the domain of interpersonal relationships, relationships with faculty (2.08) was the area with the highest mean stress score. The mean score for growth mindset was 3.10 out of 5 points. The mean score for the subdomain of intelligence was 3.20, and that for the subdomain of personality was 3.00. The mean score for adjustment to college life was 3.40 out of 5 points. The subdomain with the highest mean score was academic activities (3.94) and that with the lowest mean score was career preparation (2.98) (Table 2).

**Table 2:** Levels of college life stress, growth mindset, and adjustment to college life (*N *= 250).

Variables	Item	M ± SD	Min	Max	Range
**College life stress**	50	**2.36 ± 0.57**	1.20	4.38	1 ~ 5
Interpersonal relationships	23	1.79 ± 0.62	1.00	4.33	
Relationships with friend	5	1.62 ± 0.82	1.00	4.80	
Relationships with lover	6	1.72 ± 0.81	1.00	4.33	
Relationships with family	6	1.73 ± 0.78	1.00	5.00	
Relationships with faculty	6	2.08 ± 0.87	1.00	5.00	
Task-related stress	27	2.81 ± 0.67	1.23	4.57	
Academic problems	7	3.55 ± 0.84	1.14	5.00	
Economic problems	7	2.13 ± 0.91	1.00	5.00	
Future problems	8	2.97 ± 0.82	1.00	5.00	
Value problems	5	2.60 ± 0.92	1.00	5.00	
**Growth mindset**	8	**3.10 ± 0.71**	1.13	4.75	1 ~ 5
Intelligence	4	3.20 ± 0.85	1.00	5.00	
Personality	4	3.00 ± 0.85	1.25	5.00	
**Adjustment to college life**	19	**3.40 ± 0.57**	1.84	4.84	1 ~ 5
Interpersonal relationships	4	3.13 ± 0.83	1.00	5.00	
Academic activities	4	3.94 ± 0.58	2.25	5.00	
Career preparation	4	2.98 ± 0.87	1.00	5.00	
Personal psychology	4	3.76 ± 0.77	1.75	5.00	
Social experiences	3	3.13 ± 0.97	1.00	5.00	

M=mean; SD=standard deviation.

### Differences in college life stress, growth mindset, and adjustment to college life by characteristics including demographics

College life stress was significantly associated with perceived health status and satisfaction with the nursing major. Participants who perceived their health status as good or very good (χ^2^ = 24.75, *p*=<.001) and were satisfied or very satisfied with the nursing major (F = 13.32, *p*=<.001) showed significantly lower levels of college life stress than did others. There was no association between college life stress and other characteristics including gender, grade, religion, type of residence, reason for majoring in nursing, focus of high school curriculum, and club activities (Table 1).

There were no significant associations between growth mindset and characteristics including gender, grade, religion, type of residence, perceived health status, reason for majoring in nursing, satisfaction with nursing major, focus of high school curriculum, and club activities. By type of growth mindset, both growth mindset of intelligence and growth mindset of personality did not show significant associations with characteristics including demographics (Table 1).

Adjustment to college life was significantly associated with grade, religion, club activities, perceived health status, and satisfaction with the nursing major. Higher levels of adjustment to college life were reported in those in their senior year (F = 7.98, *p*=<.001), with a religion (t = 2.24, *p* = .026), and participating in club activities (t = 3.96, *p*=<.001). Participants who perceived their health status as good or very good showed higher levels of adjustment to college life than those who perceived their health status as poor (F = 7.02, *p*=<.001). In terms of satisfaction with the nursing major, the lowest level of adjustment to college life was reported in nursing students who were not satisfied at all with their major (F = 16.48, *p*=<.001). There was no association between adjustment to college life and other characteristics including gender, type of residence, reason for majoring in nursing, and focus of high school curriculum (Table 1).

### Correlations among variables

In the correlation analysis, adjustment to college life had a moderate negative correlation with college life stress (r = −.37, *p*=<.001) and had a low positive correlation with growth mindset (r = .27, *p*=<.001). College life stress had a low negative correlation with growth mindset (r = −.23, *p*=<.001) (Table 3). The results of the correlation analysis according to the types of growth mindsets showed that adjustment to college life showed weak positive correlations with growth mindset of intelligence (r = .22, *p*=<.001) and growth mindset of personality (r = .23 *p*=<.001). College life stress also showed weak negative correlations with growth mindset of intelligence (r = −.17, *p*=<.001) and growth mindset of personality (r = −.21, *p*=<.001).

**Table 3:** Correlations among college life stress, growth mindset and adjustment to college life (*N *= 250).

Variables	College life stress	Growth mindset	Adjustment to college life
	r (*p*)	r (*p*)	r (*p*)
College life stress	1		
Growth mindset	−.23 (<.001)	1	
Adjustment to college life	−.37 (<.001)	.27 (<.001)	1

### The mediating effect of growth mindset on the relationship between college life stress and adjustment to college life

In the hierarchical regression equation, the assumptions of normality, linearity, variance equality, and multi-collinearity were confirmed via residual analysis. Characteristics including demographics that showed significant associations with adjustment to college life in the univariate analysis, were entered into the mediation model as covariates. The mediating effect of growth mindset in the relationship between college life stress and adjustment to college life is shown in Table 4.

**Table 4:** Mediation effect analysis of growth mindset in the relationship between college life stress and adjustment to college life (*N* = 250).

Independent variable	Dependent variable	B		SE	*t*		*p*	95% CI		
								LLCI		ULCI
College Life Stress	→ Growth Mindset	−.04		.01	−3.12		.002	−.071		−.016
College Life Stress	→ Adjustment to College Life	−.05		.02	−2.43		.016	−.098		−.010
Growth Mindset		.33		.10	3.28		.001	.131		.527
**effect**	**Effect**		**Boot SE**			**95% CI**				
						**Boot LLCI**			**Boot ULCI**	
Total effect	−.07		.02			−.112			−.024	
Direct effect	−.05		.02			−.098			−.010	
Indirect effect	−.01		.01			−.030			−.003	

CI = confidence interval; LLCI = lower level confidence interval; SE = standard error; ULCI = upper level confidence interval.

**Fig 1:**
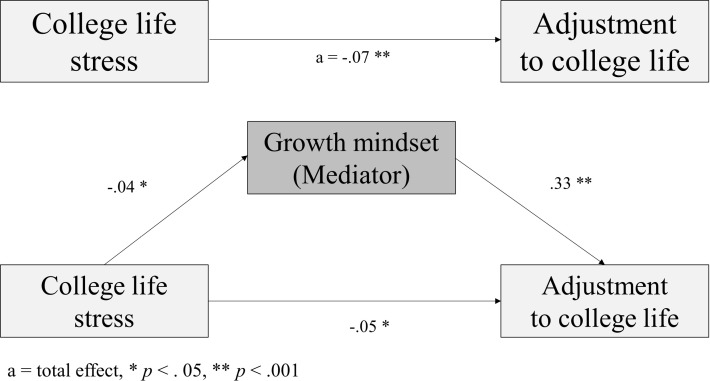
Mediating effect of growth mindset using the PROCESS Macro.

As the first step, the impact of college life stress on growth mindset, the mediating variable, was examined. It was found that college life stress had a significant negative but small impact on growth mindset after controlling for covariates (B = −0.04, *p* = .002). In the next step, the influence of college life stress and growth mindset on adjustment to college life was tested. After controlling for covariates, the regression analysis showed that college life stress had a significant small direct effect on adjustment to college life (B = −0.05, *p* = .016) and that growth mindset had a significant positive moderate effect on adjustment to college life (B = 0.33, *p* = . 001). In the analysis of the indirect effect of the mediating variable using bootstrapping analysis, the bias that corrected a bootstrap 95% CI (confidence interval) for growth mindset did not include zero by having a lower limit of −0.030 and an upper limit of −0.003, verifying the mediating effect of growth mindset (B = −0.01) (Fig 1).

To further examine which type of growth mindset mediates the relationship between college life stress and adjustment to college life, the mediating effects of growth mindset of intelligence and growth mindset of personality were analyzed separately. It was found that both growth mindset of intelligence and growth mindset of personality had mediating effects in the relationship between college life stress and adjustment to college life. The results are presented in Tables 5 and 6.

**Table 5:** Mediation effect analysis of growth mindset of intelligence in the relationship between college life stress and adjustment to college life (*N* = 250).

Independent variable	Dependent variable		B		SE	*t*		*p*	95% CI		
									LLCI		ULCI
College Life Stress	→ Growth Mindset of Intelligence		−.02		.01	−2.02		.045	−.033		−.0004
College Life Stress	→ Adjustment to College Life		−.06		.02	−2.73		.001	−.104		−.017
Growth Mindset of Intelligence			.48		.17	2.83		.005	.145		.810
**effect**		**Effect**		**Boot SE**			**95% CI**				
							**Boot LLCI**			**Boot ULCI**	
Total effect		−.07		.02			−.112			−.024	
Direct effect		−.06		.02			−.104			−.017	
Indirect effect		−.01		.01			−.021			−.0003	

CI = confidence interval; LLCI = lower level confidence interval; SE = standard error; ULCI = upper level confidence interval.

**Table 6:** Mediation effect analysis of growth mindset of personality in the relationship between college life stress and adjustment to college life (*N* = 250).

Independent variable	Dependent variable		B		SE	*t*		*p*	95% CI		
									LLCI		ULCI
College Life Stress	→ Growth Mindset of Personality		−.03		.01	−3.22		.001	−.043		−.010
College Life Stress	→ Adjustment to College Life		−.06		.02	−2.52		.012	−.100		−.012
Growth Mindset of Personality			.44		.17	2.63		.009	.111		.778
**effect**		**Effect**		**Boot SE**			**95% CI**				
							**Boot LLCI**			**Boot ULCI**	
Total effect		−.07		.02			−.112			−.025	
Direct effect		−.06		.02			−.100			−.012	
Indirect effect		−.01		.01			−.026			−.002	

CI = confidence interval; LLCI = lower level confidence interval; SE = standard error; ULCI = upper level confidence interval.

## Discussion

This study was conducted to investigate the mediating role of growth mindset on the relationships between college life stress and adjustment to college life among nursing students. The hypothesis of this study was supported, as higher levels of college life stress hindered nursing students’ adjustment to college life, and growth mindset was a mediating variable in this relationship, thereby enhancing their adjustment to college life.

In this study, nursing students’ college life stress was found to be at a moderate level, which is somewhat higher than those reported in previous studies on general college students that used an identical scale to the current study [38,39]. This finding suggests that nursing students may experience stress and aligns with the view that college life for nursing students is stressful [8]. In this study, nursing students with better perceived health status and higher satisfaction with their nursing major exhibited lower levels of college life stress. These findings are consistent with previous studies indicating that perceived health status and major satisfaction are significant factors influencing college life stress [9,40]. This highlights the importance of considering these factors when developing strategies to reduce stress among nursing students. Specifically, interventions aimed at improving students’ health status, such as promoting physical activity, balanced nutrition, and mental health support, could effectively mitigate stress levels. Similarly, fostering satisfaction with the nursing major through tailored academic support and career counseling may further contribute to reducing stress.

In this study, nursing students’ growth mindset was found to be at a moderate level. The results showed that there were no significant differences in growth mindset among nursing students according to characteristics including demographics, which is inconsistent with the finding of previous research [41] that reported significant differences in growth mindset levels based on demographic characteristics. There were also no associations between characteristics including demographics and both types of growth mindset. This discrepancy may be attributed to the growth mindset measurement tool used in this study, which comprised only four items each for intelligence and personality growth mindsets, possibly rendering it insufficiently sensitive. It is recommended that future studies employ a measurement tool with a greater number of items and a higher level of sensitivity to assess growth mindset more comprehensively.

In this study, adjustment to college life of nursing students was moderate, which is similar to the results of previous studies of nursing students [42] and students with other majors [30,43] that used an identical scale to this study. The levels of adjustment to college life in this study were higher among nursing students reporting better health status, which is consistent with the findings of previous research [44]. Adjustment to college life was also higher among those who were more satisfied with the nursing major than those who were not, supporting the view that college students’ satisfaction with their major affects their adjustment at college [42]. In this study, over half of the nursing students (61.2%) expressed satisfaction with the nursing major, showing that nursing students are generally satisfied with their major. As high levels of satisfaction with the major were associated with adjustment to college life in this study, the importance of satisfaction with the major should be recognized and considered by the nursing college.

Significant differences in the level of adjustment to college life were also observed according to grade, religion, and participation in club activities, supporting the findings of previous research [13,32]. The increase in adjustment to college life with higher grades in this study may be attributed to students’ accumulated experience and understanding of college life, which enhances their ability to adapt to the campus environment and academic demands. This is consistent with the previous view that as students progress through grades, they develop stress management and coping strategies, build confidence, and adapt more effectively to academic and social environments [45]. Pargament’s (1997) theory of religious coping explains how individuals utilize religious beliefs and behaviors to resolve problems or adapt in stressful situations [46]. Religious coping is classified into positive (e.g., collaborating with God to solve problems, seeking spiritual support) and negative (e.g., interpreting stress as divine punishment, feeling abandoned by God) strategies. Positive religious coping is associated with better psychological adjustment and higher levels of well-being [47]. The finding that nursing students with religious affiliations showed higher levels of adjustment to college life in this study suggests that they may have adapted more effectively to stress through positive religious coping strategies. The higher level of adjustment to college life observed among nursing students who participated in club activities supports previous research suggesting that club involvement contributes to the formation of social networks, provides emotional support that reduces stress [48], and offers informational support that alleviates academic and life stress, thereby playing an important role in facilitating college adjustment [49].

In the correlation analysis, increased college life stress was associated with decreased adjustment to college life, which supports the hypothesis of this study and is consistent with the findings of previous research on nursing students [50,51]. The magnitude of the correlation was 0.37, which corresponds to a medium effect size according to Cohen’s criteria [52]. While the relationship between college life stress and adjustment to college life was not particularly strong, it was meaningful, suggesting that stress management may play a crucial role in enhancing nursing students’ adjustment to college life. These results highlight the importance of interventions aimed at reducing college life stress as an effective strategy to improve adjustment among nursing students.

In this study, increased growth mindset was correlated with increased adjustment to college life, supporting the hypothesis of this study. The correlation coefficient between growth mindset and adjustment was relatively small (0.27), indicating a weak positive relationship. The correlation analysis also showed that increased college life stress was associated with decreased growth mindset, supporting the hypothesis of this study. The correlation coefficient between college life stress and growth mindset was relatively small (0.23), indicating a weak negative relationship. This result may reflect the complex and multifactorial nature of psychological stress and cognitive traits such as growth mindset, where other mediating factors could influence the observed relationship. These findings align with previous meta-analysis research that reports the negative link of psychological distress to growth mindset [27]. Furthermore, the findings also support the premise that growth mindset is negatively associated with psychological stress among college students [53] and clinical nurses [54].

In this study, it was hypothized that growth mind plays a mediating role between nursing student’s college life stress and adjustment to college life. According to the study results, the mediating role of growth mindset was identified in the relationship between nursing students’ college life stress and adjustment to college life. This suggests that while higher college life stress may be associated with lower adjustment levels, fostering a growth mindset could mitigate these negative effects and enhance adaptation to college life. These findings highlight the importance of growth mindset on inducing the wellbeing of nursing students, and suggest the growth mindset concept as a potential intervention for accommodating students’ adjustment in college. Although the mediating effect of growth mindset in the relationship between college life stress and adjustment to college life was statistically significant, the magnitude of the indirect effect was relatively small (B = −0.01). This modest effect size is primarily attributed to the small impact of college life stress on growth mindset, despite the relatively strong association between growth mindset and adjustment. Notably, the magnitude of the effect from growth mindset to adjustment (B = 0.33) is considerably larger than that of the effect from college life stress to growth mindset (B = −.04), indicating that growth mindset plays a particularly critical role in facilitating adjustment to college life. Future studies may consider exploring additional mediating or moderating variables—such as coping styles, resilience, or social support—that could operate in conjunction with growth mindset to more effectively mitigate the effects of stress.

The analysis of mediating effects also revealed that both growth mindset of intelligence and growth mindset of personality mediated the relationship between college life stress and adjustment to college life. This finding is consistent with previous studies showing that SSIs targeting growth mindset of intelligence reduce stress and enhance academic achievement and psychological well-being [19]. It also aligns with previous findings that SSIs targeting growth mindset of personality improve adolescents’ stress recovery, reduce depressive symptoms, and enhance behavioral and emotional control [28]. Therefore, higher education institutions can utilize integrated growth mindset interventions that simultaneously target growth mindsets of both intelligence and personality in order to reduce college life stress among nursing students and help them better achieve adjustment to college life. Furthermore, it is suggested that these interventions be extended to students in other majors who experience high levels of stress during their college life.

This study had some limitations. Causality is difficult to be established in this study owing to the cross-sectional study design. Selection bias may have occurred because the participants were recruited based on a convenience sampling method. Because the participants of the study were a group of nursing students in South Korea, the results cannot be generalized to nursing students in other countries. Nevertheless, this study contributes to knowledge on nursing education by addressing the adjustment to college life of nursing students, an important learner group in the nursing field. Furthermore, it provides a theoretical basis for the development of interventions focusing on growth mindset to enhance adjustment to college life of nursing students.

## Conclusion

In this study, higher levels of adjustment to college life were reported in nursing students who were in good health status and were satisfied with the nursing major. Increased levels of adjustment to college life were associated with decreased levels of college life stress and increased levels of growth mindset in nursing students. Growth mindset mediated the link between college life stress and adjustment to college life of nursing students. Further research should focus on identifying other interpersonal and environmental correlates of adjustment to college life of nursing students and examine the effects of various interventions for enhancing growth mindset of intelligence and personality, which can ultimately improve their adjustment to college life.

## Supporting information

S1 Table Correlations among college life stress, growth mindset of intelligence, and adjustment to college life (*N* = 250). (DOCX)

S2 Table Correlations among college life stress, growth mindset of personality, and adjustment to college life (*N* = 250). (DOCX)

S3 File Dataset. (XLSX)
