# Supplementary material for: Mediating role of growth mindset between college life stress and adjustment to college life among nursing students: A-cross sectional study
Source: PLoS One. 2025 Jun 25;20(6):e0325774. doi: 10.1371/journal.pone.0325774 (PMC12192134; doi:10.1371/journal.pone.0325774)
Supplement: S1 Table — (DOCX) [file pone.0325774.s001.docx]

**S1 Table. Correlations among college life stress, growth mindset of intelligence, and adjustment to college life (*N*=250).**

| Variables | College life stress | Growth mindset of intelligence | Adjustment to  college life |
| --- | --- | --- | --- |
|  | r (*p*) | r (*p*) | r (*p*) |
| College life stress | 1 |  |  |
| Growth mindset of intelligence | -.17 (<.001) | 1 |  |
| Adjustment to  college life | -.37 (<.001) | .22 (<.001) | 1 |
